# BMC Biomedical Engineering: a home for all biomedical engineering research

**DOI:** 10.1186/s42490-019-0004-1

**Published:** 2019-01-30

**Authors:** Alexandros Houssein, Alan Kawarai Lefor, Antonio Veloso, Zhi Yang, Jong Chul Ye, Dimitrios I. Zeugolis, Sang Yup Lee

**Affiliations:** 1 Springer Nature, 4 Crinan Street, London, N1 9XW UK; 2 Department of Surgery, Jichi Medical University, Shimotsuke, Tochigi Japan; 3 Laboratory of Biomechanics and Functional Morphology, Faculty of Human Kinetics, Lisbon, Portugal; 4 Department of Biomedical Engineering, University of Minnesota, Minneapolis, MN USA; 5 Department of Bio and Brain Engineering, Korea Advanced Institute of Science and Technology, Daejeon, Republic of Korea; 6 Regenerative, Modular and Developmental Engineering Laboratory (REMODEL), Biomedical Sciences Building, National University of Ireland Galway (NUI Galway), Galway, Ireland; 7 Department of Chemical and Biomolecular Engineering, Korea Advanced Institute of Science and Technology, Daejeon, Republic of Korea

## Abstract

This editorial accompanies the launch of *BMC Biomedical Engineering*, a new open access, peer-reviewed journal within the BMC series, which seeks to publish articles on all aspects of biomedical engineering. As one of the first engineering journals within the BMC series portfolio, it will support and complement existing biomedical communities, but at the same time, it will provide an open access home for engineering research. By publishing original research, methodology, database, software and review articles, *BMC Biomedical Engineering* will disseminate quality research, with a focus on studies that further the understanding of human disease and that contribute towards the improvement of human health.

## Introduction

Biomedical engineering is a multidisciplinary field that integrates principles from engineering, physical sciences, mathematics and informatics for the study of biology and medicine, with the ultimate goal of improving human health and quality of life.

Biomedical engineering is not a new concept; however, it was not until the 1900s when rapid technological advancements in the chemical, physical and life sciences influenced breakthroughs in the prevention, diagnosis and treatment of disease. The invention of the electrocardiograph, the concept of x-ray imaging, the electron microscope, the mechanical heart valve and human genome sequencing, are just a few examples of technological innovations that revolutionised science and medicine and changed the approach to human healthcare. Current biomedical engineering technologies are a growing part of clinical decision making, which can now be influenced from multiscale observations, ranging from the nano to the macro-scale.

Today, the need for innovation in health technologies is ever more prominent. The annual global healthcare spending has seen continued growth and is projected to reach a staggering $8.7 trillion by 2020 [1]. Global health challenges are becoming more complex, wide spread and difficult to control. Resources are scarce and with a growing population, our society has a need for affordable, portable and sustainable solutions. The World Health Organisation has pledged to make a billion lives healthier by 2023 [2], a goal that will require widespread commitment by governments, funding agencies, researchers and clinicians. Biomedical engineers will be at the heart of this movement and face a responsibility for continuous innovation. Biomedical engineering research is expected to create health technologies that will drastically improve the prevention, diagnosis and treatment of disease, as well as patient rehabilitation. As an example, the NIH 2016–2020 strategic plan focuses on point of care and precision medicine technologies including genetic engineering, microfluidics, nanomedicine, imaging, digital/mobile-Health and big data [3].

*BMC Biomedical Engineering* will strive to complement these efforts and provide an open access venue for the dissemination of all biomedical engineering research. As part of the BMC series, a portfolio of journals serving communities across all sciences, the Journal will act as a resource for a wide range of disciplines. It aims to support scientists, engineers and clinicians by making their research openly and permanently available, irrespective of their location or affiliation.

## Aims and scope

*BMC Biomedical Engineering* considers articles on all aspects of biomedical engineering, including fundamental, translational and clinical research. It combines tools and methods from biology and medicine with mathematics, physical sciences and engineering towards the understanding of human biology and disease and the improvement of human health. The Journal will publish a range of article types, including research, methodology, software, database and review articles.

As part of the BMC series, a collection of open access, peer-reviewed and community focused journals covering all areas of science, editorial decisions will not be made on the basis of the interest of a study or its likely impact. Studies must be scientifically valid. For research articles this includes a scientifically sound research question, the use of suitable methods and analysis, and following community-agreed standards relevant to the research field.

*BMC Biomedical Engineering* aims to publish work that undergoes a thorough peer review process by appropriate peer-reviewers and is deemed to be a coherent and valid addition to the scientific knowledge. It aims to provide an open access venue which allows for immediate and effective dissemination of research and enables our readers to explore and understand the latest developments, trends and practices in biomedical engineering. We believe that open access and the Creative Commons Attribution License [4] are essential to this, allowing universal and free access to all articles published in the Journal and allowing them to be read and the data re-used without restrictions. *BMC Biomedical Engineering* will work closely with the rest of the journals in the BMC series portfolio [5] to help authors find the right home for their research. We will highlight selected journal content through various promotional channels to ensure the research reaches its target audience and receives the attention it deserves.

## Editorial sections

Many new technologies that have revolutionised biomedical engineering require the coalition of previously independent communities. 3D bioprinting of tissues and organs brings together methods from cell biology, biomaterials, nanotechnology and engineering and is being used for the transplantation of tissues, including skin, bone, muscle, soft tissue, cartilage and others [6, 7]. The concept of tissue and disease modelling is being driven towards drug discovery and toxicology studies, aiming to increase the yield of drug testing by tackling limitations of current cell and animal models [8].

New approaches in natural and synthetic biomaterials have redefined bioelectronics. Silk fibroins and other unconventional interfaces can form flexible electronics and challenge the use of silicon-based technologies. For biomedical applications, these new approaches present advantages not only due to their biocompatibility and low cost, but also for their electromechanical and optical virtues [9]. Implantable probes are being redesigned so that they facilitate long term stability and high resolution, without perturbing the biological system or creating an immune response. Such technologies are now able to facilitate recordings of single neurons in vivo, in a chronically stable manner, with applications to the restoration of vision and retinal prosthetics [10].

For many years biomedical imaging has been connecting microscopic discoveries with macroscopic observations. Photoacoustic tomography (PAT) is now able to image large spatial scales, from organelles to small animals, at very high speeds [11]. In fact, single-shot real-time imaging can operate at 10 trillion frames per second and is finding applications in breast cancer diagnosis [12, 13].

In the field of medical robotics, new approaches combine machine learning and artificial intelligence to strengthen the clinician’s decision making. Others are leveraging augmented reality (AR) to facilitate better immersion and more natural surgical workflows for computer assisted orthopaedic surgery [14].

*BMC Biomedical Engineering* celebrates the interdisciplinary nature of the field. In order to navigate the wide range of biomedical engineering research, the Journal is structured in six editorial sections.

- Biomaterials, nanomedicine and tissue engineering
- Medical technologies, robotics and rehabilitation engineering
- Biosensors and bioelectronics
- Computational and systems biology
- Biomechanics
- Biomedical Imaging

We are delighted to welcome our founding Section Editors along with a growing international group of Editorial board Members [15, 16]. The Journal is supported by an expert Editorial Advisory group of senior engineers and scientists, which is chaired by Distinguished Professor Sang Yup Lee. Together with the in-house Editor, this group will provide academic leadership and expertise and will work together to transverse into multiple clinical and engineering disciplines. The Editorial Board will keep growing and developing to reflect and adapt to the nature of this diverse community.

### Biomaterials, nanomedicine and tissue engineering section

This section primarily focuses on the development of biofunctional tissue substitutes, which possess the highest level of biomimicry, through recapitulation of nature’s innate sophistication and thorough processes. It considers research, methods, clinical trials, leading opinion and review articles on the development, characterisation and application of nano- and micro- biofunctional biomaterials, cell-assembled tissue substitutes, diagnostic tools, microfluidic devices and drug/gene discovery and delivery methods. Manuscripts focusing on permanently differentiated, engineered and stem cell biology and application are welcome. This section will place a substantial focus on clinical translation and technologies that advance the current status-quo. As such, articles that enhance the scalability and robustness of tissue engineering methodologies, or that enable new and improved industrial or clinical applications of biomedical engineering discoveries, tools and technologies are strongly encouraged.

### Medical technologies, robotics and rehabilitation engineering section

This section seeks to represent research in engineering that encompasses a wide range of interests across medical specialties, including orthopaedic, cardiovascular, musculoskeletal, craniofacial, neurological, urologic and other medical technologies. It will consider research on medical robotics, computer assisted technologies, medical devices, e/m-health and other medical instrumentation. It aims to improve the prevention, diagnosis, intervention and treatment of injury or disease and it welcomes articles that represent new approaches to engineering that may be useful in the care of patients. Technical and practical aspects of rehabilitation engineering, from concept to clinic and papers on improving the quality of life of patients with a disability are encouraged. The section also seeks to represent clinically important research that is based on new and emerging technologies. This could include clinical studies of new approaches to robotic-assisted surgery, clinical studies of new devices, or other studies that are close to patient care or rehabilitation.

### Biosensors and bioelectronics section

This section considers articles on the theory, design, development and application on all aspects of biosensing and bioelectronics technologies. The section will consider approaches that combine biology and medicine with sensing and circuits and systems technologies on a wide variety of subjects, including lab-on-chips, microfluidic devices, biosensor interfaces, DNA chips and bioinstrumentation. It also considers articles on the development of computational algorithms (such as deep learning, reinforcement learning, etc.) that interpret the acquired signals, hardware acceleration and implementation of the algorithms, brain-inspired or brain-like computational schemes, and bioelectronics technologies that can have a wide impact in the research and clinical community. Articles on implantable and wearable electronics, low-power, wireless and miniaturised imaging systems, organic semiconductors, smart sensors and neuromorphic circuits and systems are strongly encouraged.

### Computational and systems biology section

Computational, integrative and systemic approaches are at the heart of biomedical engineering. This section considers papers on all aspects of mathematical, computational, systems and synthetic biology that result in the improvement of patient health. Integrative and multi-scale approaches, in the network and mechanism-based definition of injury and disease, or its prevention, diagnosis and treatment are welcome. Papers on high precision, interactive and personalised medicine, on digital/mobile health, on complex/big data analytics and machine learning, or on systemic and informatics approaches in a healthcare or clinical setting are encouraged.

### Biomechanics section

This section represents the interdisciplinary field of biomechanics and investigates the relationship of structure with function in biological systems from the micro- to the macro- world. It considers papers on all aspects of analytical and applied biomechanics at all scales of observation, that improve the diagnosis, therapy and rehabilitation of patients or that advance their kinetic performance. The topics of interest range from mechanobiology and cell biomechanics to clinical biomechanics, orthopaedic biomechanics and human kinetics. Articles on the mechanics and wear of bones and joints, artificial prostheses, body-device interaction, musculoskeletal modelling biomechanics and solid/fluid computational approaches are strongly encouraged.

### Biomedical imaging section

Biomedical imaging has been connecting microscopic discoveries with macroscopic observations for the diagnosis and treatment of disease and has seen considerable advances in recent years. This section will consider articles on all biomedical imaging modalities including medical imaging (MRI, CT, PET, ultrasound, x-ray, EEG/MEG), bio-imaging (microscopy, optical imaging) and neuroimaging across all scales of observation. Its primary focus will be to foster integrative approaches that combine techniques in biology, medicine, mathematics, computation, hardware development and image processing. Articles on new methodologies or on technical perspectives involving novel imaging concepts and reconstruction methods, machine learning, sparse sampling and statistical analysis tool development are encouraged.

## Conclusion

The motivation for the launch of *BMC Biomedical Engineering* is to create an authoritative, unbiased and community-focused open access journal. We are committed to working together with our authors, editors and reviewers to provide an inclusive platform for the publication of high-quality manuscripts that span all aspects of biomedical engineering research. We welcome articles from all over the world and we will devote our efforts to ensure a robust and fair peer-review process for all. We believe in continuous improvement and we encourage the community to get in touch with us to provide ideas and feedback on how to improve the Journal and serve the community better.

We hope you will find the first group of articles an interesting and valuable read, and we look forward to working with you all to disseminate research into the exciting field of biomedical engineering.

## Abbreviations

AR: Augmented Reality
CT: Computed Tomography
EEG: Electroencephalogram
MEG: Magnetoencephalography
PAT: Photoacoustic Tomography
PET: Positron Emission Tomography
